# Weighted-Support Vector Machine Learning Classifier of Circulating Cytokine Biomarkers to Predict Radiation-Induced Lung Fibrosis in Non-Small-Cell Lung Cancer Patients

**DOI:** 10.3389/fonc.2020.601979

**Published:** 2021-02-01

**Authors:** Hao Yu, Ka-On Lam, Huanmei Wu, Michael Green, Weili Wang, Jian-Yue Jin, Chen Hu, Shruti Jolly, Yang Wang, Feng-Ming Spring Kong

**Affiliations:** ^1^ Biomedical Engineering, Shenzhen Polytechnic, Shenzhen, China; ^2^ BioHealth Informatics, School of Informatics and Computing, Indiana University - Purdue University Indianapolis (IUPUI), Indianapolis, IN, United States; ^3^ Department of Clinical Oncology, Li Ka Shing (LKS) Faculty of Medicine, The University of Hong Kong, Hong Kong, Hong Kong; ^4^ Clinical Oncology Center, The University of Hong Kong-Shenzhen Hospital, Shenzhen, China; ^5^ Radiation Oncology, Ann Arbor VA Health Care, Ann Arbor, MI, United States; ^6^ Radiation Oncology, University of Michigan, Ann Arbor, MI, United States; ^7^ University Hospitals, Cleveland Medical Center, Seidman Cancer Center and Case Comprehensive Cancer Center, Case Western Reserve University, Cleveland, OH, United States; ^8^ Sidney Kimmel Comprehensive Cancer Center, Johns Hopkins University School of Medicine, Baltimore, MD, United States

**Keywords:** Support Vector Machine, radiation-induced lung fibrosis, non-small-cell lung cancer, cytokine, lung dosimetric factors

## Abstract

**Background:**

Radiation-induced lung fibrosis (RILF) is an important late toxicity in patients with non-small-cell lung cancer (NSCLC) after radiotherapy (RT). Clinically significant RILF can impact quality of life and/or cause non-cancer related death. This study aimed to determine whether pre-treatment plasma cytokine levels have a significant effect on the risk of RILF and investigate the abilities of machine learning algorithms for risk prediction.

**Methods:**

This is a secondary analysis of prospective studies from two academic cancer centers. The primary endpoint was grade≥2 (RILF2), classified according to a system consistent with the consensus recommendation of an expert panel of the AAPM task for normal tissue toxicity. Eligible patients must have at least 6 months’ follow-up after radiotherapy commencement. Baseline levels of 30 cytokines, dosimetric, and clinical characteristics were analyzed. Support vector machine (SVM) algorithm was applied for model development. Data from one center was used for model training and development; and data of another center was applied as an independent external validation.

**Results:**

There were 57 and 37 eligible patients in training and validation datasets, with 14 and 16.2% RILF2, respectively. Of the 30 plasma cytokines evaluated, SVM identified baseline circulating CCL4 as the most significant cytokine associated with RILF2 risk in both datasets (*P* = 0.003 and 0.07, for training and test sets, respectively). An SVM classifier predictive of RILF2 was generated in Cohort 1 with CCL4, mean lung dose (MLD) and chemotherapy as key model features. This classifier was validated in Cohort 2 with accuracy of 0.757 and area under the curve (AUC) of 0.855.

**Conclusions:**

Using machine learning, this study constructed and validated a weighted-SVM classifier incorporating circulating CCL4 levels with significant dosimetric and clinical parameters which predicts RILF2 risk with a reasonable accuracy. Further study with larger sample size is needed to validate the role of CCL4, and this SVM classifier in RILF2.

## Introduction

Lung cancer is the leading cause of cancer-related death. Non-small-cell lung cancer (NSCLC) is the predominant (85%) form of lung cancer (1). The majority of patients with locally advanced NSCLC are unresectable and treated with chemotherapy and radiation therapy (RT). While curative in a subset of patients, RT as a mainstay local treatment of cure for NSCLC is often limited by the concerns of radiation-induced lung toxicities (RILT), including radiation pneumonitis (RP) and radiation-induced lung fibrosis (RILF) (2). The risk of RP following receipt of thoracic RT has been widely studied (3). However, RILF as another important RILT whose significance was recently highlighted in a global context recently by a global workshop organized by the Center for Cancer Research of the National Cancer Institute (NCI) global workshop (4) has not been adequately reported.

RILF is typically considered to be a late and irreversible pathologic process (5, 6). Persistent injury of type II alveolar epithelial cells, infiltration of inflammatory cells, deposition of collagen, and formation of lung fibrosis (7, 8) are contributing pathophysiologic mechanisms in RILF. Clinically, it can cause dyspnea, impaired lung function, and even fatal respiratory insufficiency (8–10). Clinically significant RILF affects quality of life and can be a critical condition for long-term survivors (10). Unfortunately, RILF remains understudied, and the treatment is primarily supportive with supplemental oxygen for symptomatic relief (11). Thus, it is crucial to identify risk factors and models that may predict RILF prior to treatment with RT.

Multiple studies have identified dosimetric correlates of RILF (8, 12–14), but models which integrate clinical, biological, and dosimetric features to predict RILF have not been constructed. Recently, urine gastrin-releasing peptide (GRP) (15), serum club cell secretory protein (CCSP), and serum surfactant protein D (SP-D) (16) levels were found to predict RILF development in mice. Cytokines play crucial roles in the interactions and communications between cells; in particular, some are essential in pathologic process of inflammation/pro-inflammation and fibrosis development, thus potentiating the effect of RP on RILF. We recently reported a significant correlation of baseline Interleukin-8 (IL-8) and C-C Motif Chemokine Ligand 2 (CCL2) levels with RP2 (RP grade≥2) risk (17). For RILF risk, we have studied the effect of circulating cytokines in mice, demonstrating that granulocyte-colony stimulating factor (G-CSF), Interleukin-6 (IL-6), and keratinocyte-derived chemokines (KCs) were significant factors (18).

In this study, with long-term follow-up data from prospective clinical trials, we hypothesized that cytokines with immunomodulating, inflammatory, and fibrosis forming effects play key roles for the development of RILF, and thus baseline cytokine levels in combination with treatment dosimetric and clinical variables can improve the predictive accuracy of RILF. Specifically, this study aimed to explore such a combined predictive model for RILF2 (RILF grade≥2) and the clinical utility of using machine learning algorithm for modeling. Weighted-Support Vector Machine (weighted-SVM) was chosen as it can handle small size and imbalanced datasets, using the weighted soft margin approach (19–21).

## Methods

### Study Population

The study population was 185 patients with NSCLC who participated in four prospective clinical trials (UMCC 2003.073, UMCC 2003.076, NCT00603057, and NCT01190527) at two Medical Centers: Cohort 1 (the Veterans Affairs Medical Center, Ann Arbor, MI) and Cohort 2 (the University of Michigan Cancer Center) from 2003 to 2016. Study eligibility included those with FDG-avid (maximum SUV ≥4.0, from PET scan of any date, any scanner); histologically or cytologically proven NSCLC; with follow-up assessment for RILF risk. All clinical data, including grading of RILF, clinical and dosimetric parameters, and blood samples, were prospectively collected. We excluded patients without follow-up and those treated with stereotactic body radiation therapy (SBRT) considering their entirely different dose fractionations and biologic mechanisms. Furthermore, all patients were required to have at least six months of follow-up for RILF, which was necessary for latency considerations.

### Radiation Treatment

All patients received daily fractionated 3D conformal radiation therapy with or without concurrent chemotherapy (Chemo). The gross tumor volume (GTV) including the primary tumor and any involved hilar or mediastinal lymph nodes was delineated on the basis of clinical, pathologic, and radiographic data which included a positron emission tomography–computed tomography (PET-CT). Radiation therapy was given in 60–86 Gy in 2–3.8 Gy fractions, including two dose escalation studies which allowed tumor prescription doses up to 86 Gy. The details and RT dose-fractionations for each trial are summarized in **Supplemental Table S1**. Since various doses/fractions were used for patients, bio-corrected radiation doses with alpha/beta = 3 were used to calculate MLD and V20 in order to compare lung biological effective dose for different RT fractionations.

### Endpoint and RILF Grading

The primary endpoint was clinical RILF grade ≥2 (RILF2). Patients were evaluated at every 3 months in year 1 and every 6 months in year 2 and every year after 3 years and after. RILF was graded prospectively, according to a predefined grading system which was consistent with the recommendation of the expert panel of an AAPM task for normal lung toxicity (22), similar to detailed statement of adverse events and radiographic changes according to CTCAE3.0 (**Table 1**). RILF2 was defined by the presence of radiologic fibrosis with dyspnea symptom (2) but without notable changes of average daily living. RILF3 was those with symptom and with changes of average daily living. RILF grade for each patient was reviewed by both American Board Certified radiologist and radiation oncologist.

**Table 1:** Diagnosis and grading for clinical radiation-induced lung fibrosis (RILF) (2, 22).

	Adverse event	Radiographic changes
Grade 1	Radiographic evidence of radiation fibrosis with no or mild dyspnea	Minimal radiographic findings (or patchy or bibasilar changes) with estimated radiographic proportion of total lung volume that is fibrotic of <25%
Grade 2	Radiation fibrosis causing dyspnea but does not interfere with ADL	Patchy or bi-basilar changes with estimated radiographic proportion of total lung volume that is fibrotic of [25%, 50%)
Grade 3	Radiation fibrosis causing dyspnea that interferes with ADL, or requiring oxygen or increase in baseline home oxygen use	Dense or widespread infiltrates/consolidation with estimated radiographic proportion of total lung volume that is fibrotic of [50%, 75%)
Grade 4	Radiation fibrosis that causes respiratory insufficiency, requires assisted ventilation	Estimated radiographic proportion of total lung volume that is fibrotic is ≥75%; honeycombing
Grade 5	Radiation fibrosis directly contributing to death	

### Plasma Cytokines

Baseline levels of 30 plasma cytokines including Epidermal growth factor (EGF), Granulocyte-colony stimulating factor (G-CSF), Granulocyte-macrophage colony stimulating factor (GM-CSF), Interferon gamma (INF-*γ*), Interleukin 10 (IL-10), Subunit beta of interleukin 12 (IL-12p40), Interleukin-12 (IL-12p70), Interleukin 13 (IL-13), Interleukin 15 (IL-15), Interleukin 17 (IL-17), Interleukin 1*α* (IL-1*α*), Interleukin 1β (IL-1*β*), Interleukin 1r*α* (IL-1r*α*), Interleukin 2 (IL-2), Interleukin 4 (IL-4), Interleukin 5 (IL-5), Interleukin 6 (IL-6), Interleukin 7 (IL-7), Interleukin 8 (IL-8), C-C motif chemokine ligand 2 (CCL2), C-C motif chemokine ligand 3 (CCL3), C-C motif chemokine ligand 4 (CCL4), C-C motif chemokine ligand 11 (CCL11), C-X-C motif chemokine ligand 10 (CXCL10), C-X3-C Motif Chemokine Ligand 1 (CX3CL1), Soluble CD40 ligand (sCD40l), Transforming growth factor-alpha (TGF-*α*), Tumor necrosis factor-*α* (TNF-*α*), Vascular endothelial growth factor (VEGF), and Transforming growth factor-beta1 TGF-*β*1 were measured. The protocol for plasma collection, storage, and cytokine measurements had been described previously (23, 24). Since the cytokine levels were right-skewed, they were normalized by a log transformation before further analysis.

### Statistical Analysis

The patients with missing data were excluded in this study. Fisher’s exact test and logistic regression were used to evaluate the statistical significance of clinical variables, dose metrics, and baseline plasma cytokine levels with RILF2. All statistical analyses were two-sided, with the overall *P* threshold of 0.05 for significance. All statistical analyses and machine learning algorithms of this study were performed using R, version 3.6.1 (25).

### Machine Learning Algorithms: Weighted-SVM Classifier

Multiple machine learning algorithms can be applied to identify significant biomarkers by building (Cohort 1) and externally validating (Cohort 2) a predictive model to classify the RILF risk. Considering the limitations of a small sample size and imbalanced datasets, the weighted-SVM algorithm was elected (21).

For SVM classifier building, limited by the sample size, only three features were allowed to avoid over-fitting. These three features were selected by machine learning algorithm to be the representative of cytokine biology, physical dosimetrics, and clinical treatment variables. The tuning hyperparameters of the weighted-SVM included the following: radial kernel, cost from 400 to 800 stepped 100, gamma from 0.001 to 0.01 stepped 0.001, and weight of cases without RILF2 from 0.1 to 0.15 stepped 0.002. The SVM classifiers with final features and hyperparameters were trained and tested in Cohort 1 by cross-validation (CV) algorithm. Five times fivefold CV algorithm was performed to control for the limited sample size. 1) The data was randomly divided into a training set and a testing set (fourfold and onefold). 2) For each set of three features and each set of hyperparameters, SVM models were generated on each training set and validated in each testing set to calculate accuracy, area under receiver operative curve (AUC), and the area under precision-recall curves (PRAUC). 3) Steps 1 and 2 were repeated five times, therefore for each set of three features and each set of hyperparameters, there were five models trained and tested; then the model closest to the mean accuracy value was chosen. 4) SVM models with different sets of three features and sets of hyperparameters were compared by accuracy, AUC and PRAUC; finally the one with the highest value was selected as the final SVM classifier.

The generalized performances of this final predicting classifier were externally validated in Cohort 2, including accuracy, sensitivity, specificity, positive predictive value (PPV), and negative predictive value (NPV), receiver operative curves (ROCs), and its corresponding AUC, and precision-recall curves and its AUC (PRAUCs). To further evaluate the prediction performance of this final SVM classifier, the generalized linear models (GLMs), which are multivariable logistic regression in binary classification, were also trained and tested in Cohort 1 by CV and externally validated in Cohort 2. The performances of SVM classifiers and GLM models were compared.

## Results

### Patient Characteristics

**Figure 1:**
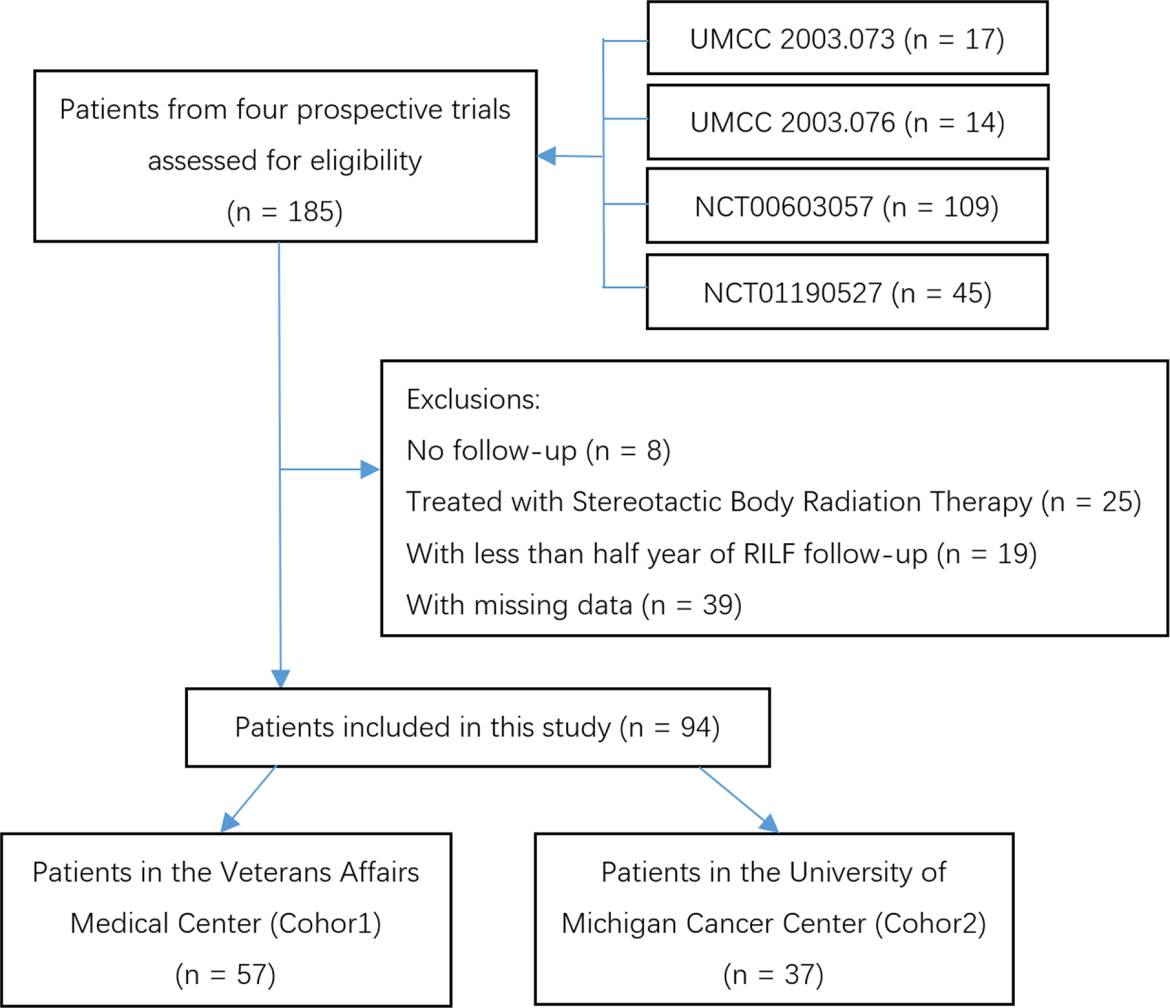
Study flow chart.

A total of 94 patients (shown in **Figure 1**) which met the analysis inclusion criteria were identified in two independent cohorts: 57 patients with eight cases of RILF2 (14%) in Cohort 1 and 37 patients with six cases of RILF2 (16.2%) in Cohort 2. The median follow-up time was 5.98 years, and the median onset time of RILF2 was 5.4 months. In Cohort 1, 56/57 patients were male with a median age of 65.7 years. Fourteen patients received RT alone, and 43 received RT with concurrent chemotherapy. In 37 patients of Cohort 2, 14/37 patients were male with a median age of 64.2 years. Six patients received RT alone, and 31 received RT with concurrent chemotherapy. Two cohorts’ clinical characteristics and dose metrics were summarized in **Table 2**.

**Table 2:** Clinical variables and dosimetric factors for radiation induced lung fibrosis (RILF).

Characteristic	Cohort 1 n (%)			Cohort 2 n (%)		
	Without RILF2 n = 49	With RILF2 n = 8	*P*	Without RILF2 n = 31	With RILF2 n = 6	*P*
Gender^$^						
Female Male	1 (2.1) 48 (97.9)	0 (0) 8 (100)	1	18 (58.1) 13 (41.9)	5 (83.3) 1 (16.7)	0.45
Age^#^						
Median 1^st^–3^rd^ Qu	65.7 60–74	67.3 58–72	0.85	65.6 57–70	60.5 59–63	0.41
KPS^#^						
Median 1^st^–3^rd^ Qu	80 80–90	90 80–90	0.62	90 80–90	90 90–90	0.62
Smoking status^$^						
Never Former Current Unknown	0 (0) 21 (42.9) 23 (46.9) 5 (10.2)	0 (0) 3 (37.5) 4 (50) 2 (12.5)	1	3 (9.6) 14 (45.2) 14 (45.2) 0 (0)	0 (0) 3 (50) 3 (50) 0 (0)	1
Chemotherapy^$^						
No Yes	14 (28.6) 35 (71.4)	0 (0) 8 (100)	0.26	6 (19.4) 25 (80.6)	0 (0) 6 (100)	0.68
Clinical staging of cancer^$^						
1 2 3	6 (12.2) 9 (18.4) 34 (69.4)	0 (0) 0 (0) 8 (100)	0.38	3 (9.7) 2 (6.5) 26 (83.8)	0 (0) 0 (0) 6 (100)	1
T stage^$^						
T1 T2 T3 T4	11 (22.4) 10 (20.4) 19 (38.8) 9 (18.4)	0 (0) 2 (25) 2 (25) 4 (50)	0.27	5 (16.1) 6 (19.4) 8 (25.8) 12 (38.7)	1 (16.7) 1 (16.7) 1 (16.7) 3 (50)	1
N stage^$^						
N0 N1 N2 N3	16 (32.7) 3 (6.1) 22 (44.9) 8 (16.3)	0 (0) 1 (12.5) 3 (37.5) 4 (50)	0.04	7 (22.6) 5 (16.1) 10 (32.3) 9 (29)	1 (16.7) 1 (16.7) 3 (50) 1 (16.7)	0.92
GTV (cm^3^)^#^						
Median 1^st^–3^rd^ Qu	89.9 48.4–234.5	272.3 192–316.2	0.09	80.6 34.8–185.3	94.7 85.1–155.3	0.76
Total Prescription Dose (Gy)^#^						
Median 1^st^–3^rd^ Qu	73.5 68.2–78.5	68.9 65.6–70	0.2	66 64.6–74.2	83.8 79.4–85.3	0.01
MLD (Gy)^#^						
Median 1^st^–3^rd^ Qu	15 12.4–17.6	15.64 14.4–16.8	0.41	13.2 10–17.2	17.4 15–18.7	0.1
V20 (%)^#^						
Median 1^st^–3^rd^ Qu	24.1 18.6–27.9	26.6 23.7–27.9	0.23	23 15.7–27.2	27.5 26.8–29.9	0.11

KPS, Karnofsky Performance Status; Chemo, concurrent chemoradiotherapy; GTV, gross tumor volume; MLD, mean lung dose.

^$^Fisher’s exact test; ^#^logistic regression; -QU = quartile.

### Univariate Analysis of Cytokines and RILF2

To explore the effect of each cytokine, conventional logistic regression was performed for each cytokine at baseline in Cohort 1. CCL4 was significantly associated with the risk of RILF2 (odds ratio, OR = 0.404, 95%CI = 0.223–0.733, *P* = 0.003). CX3CL1 level (OR = 0.494, 95%CI = 0.296–0.826, *P* = 0.007), G-CSF level (OR = 0.616, 95%CI = 0.403–0.944, *P* = 0.02), TNF-α level (OR = 0.464, 95%CI = 0.23–0.938, *P* = 0.03) and CCL11 level (OR = 0.304, 95%CI = 0.098v0.944, *P* = 0.04) were also significantly associated with the risk of RILF2.

Performing the same analysis in Cohort 2, IL-8 (OR = 0.33, 95%CI = 0.129v0.847, *P* = 0.02) and IL5 (OR = 0.613, 95%CI = 0.41–0.916, *P* = 0.02) were significantly associated with the risk of RILF2. The CCL4 level (OR = 0.536, 95%CI = 0.274–1.048, *P* = 0.07) and G-CSF level (OR = 0.668, 95%CI = 0.421–1.06, *P* = 0.09) were also borderline significant with the risk of RILF2 in Cohort 2.

### Weighted-Support Vector Machines

**Figure 2:**
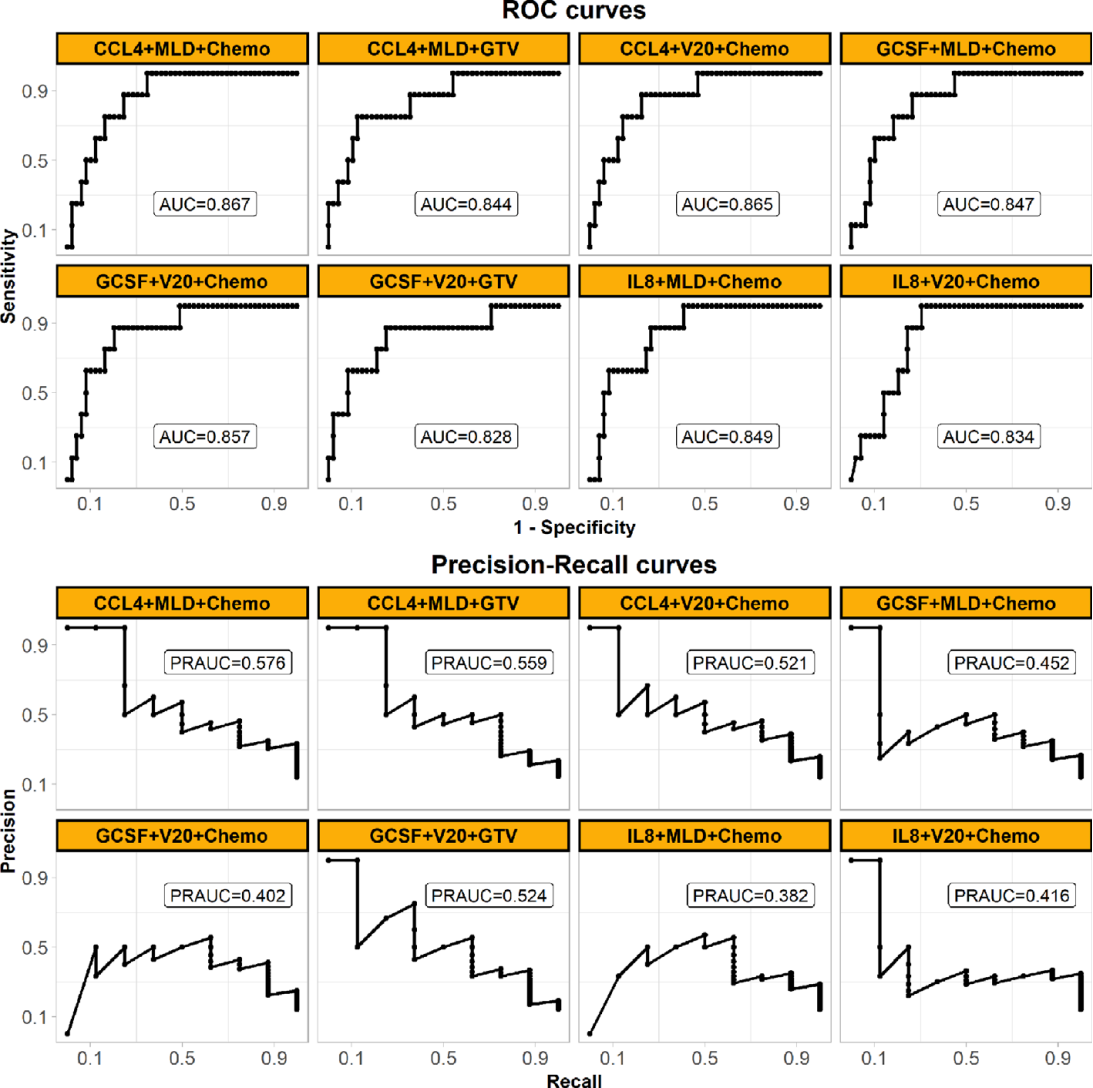
Performances of the final weighed-SVM classifier in Cohort 1 generated by machine learning algorithm comparing with the other classifiers. The panel shows their ROC (upper) and precision-recall curves (bottom) performances. The models’ features are shown in subplots’ head. The panel demonstrates that the final model of using CCL4, MLD and Chemo with the best performance. (Abbreviations: ROC, receiver operating characteristic; MLD, mean lung dose; Chemo, concurrent chemoradiotherapy; AUC, the area under the ROC curve; PRAUC, the area under the precision-recall curve).

SVM classifiers for RILF2 risk were first trained and tested in Cohort 1 by cross validation. The classifier with the best model performance was selected, and this model included three representative features from cytokines, dosimetrics, and clinical factors. For visual comparison, this model is shown in **Figure 2** to compare the model performances of other SVM classifiers with inclusion cytokines of our interest like IL-8 and most significant ones as identified above by conventional logistic regression. V20 was also included as it is commonly used in clinical practice guideline. The final weighted-SVM classifier with radial kernel included CCL4, MLD and receipt of chemotherapy as model features, while cost = 400, gamma = 0.006, and weight = 0.132 as model parameters. The classifying performance measures in Cohort 1 included: accuracy = 0.754, sensitivity = 0.875, specificity = 0.735, PPV = 0.35, NPV = 0.973, AUC = 0.867 (95% confidence interval, CI = 0.763–0.972, *P* < 0.001), PRAUC = 0.576.

**Figure 3:**
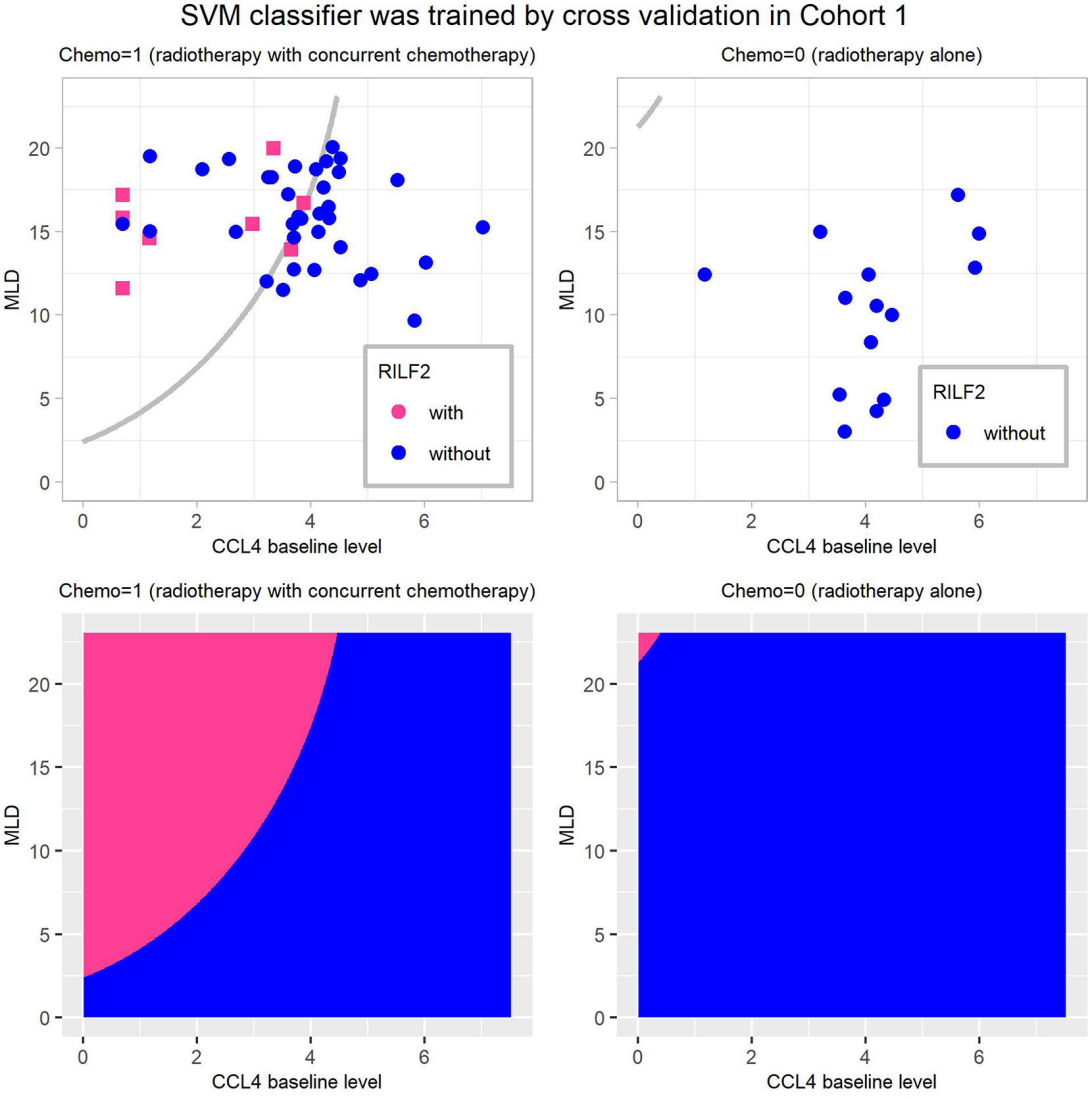
Visualization of the final weighted-SVM Classifier for RILF2 risk in Cohort 1. The decision boundaries are presented in subplots **(A, B)**, and the decision regions as subplots **(C, D)**. The decision region of the final weighted-SVM classifier with radial kernel, include three features: CCL4, MLD, Chemo, using parameters as: cost = 400, gamma = 0.006, and weight = 0.132. CCL4 is in log transformation of pg/ml. The subplots **(A, C)** are for patients treated with radiation therapy with concurrent chemotherapy (Chemo = 1) and the subplots **(B, D)** are for patients treated with radiotherapy alone (Chemo = 0). In these subplots, without RILF2, are defined as blue and with RILF2 are shown in red. SVM, Support Vector Machine.

**Figure 3** shows the visualization of this final weighted-SVM classifier for Cohort 1 patients. In patients who received chemotherapy, 7/8 patients actually had RILF2 when they were predicted to have RILF2, while 36/49 patients without RILF2 were predicted to not develop RILF2. In patients who received radiation alone, this final SVM model was classified correctly in all patients 100%.

### External Validation

**Figure 4:**
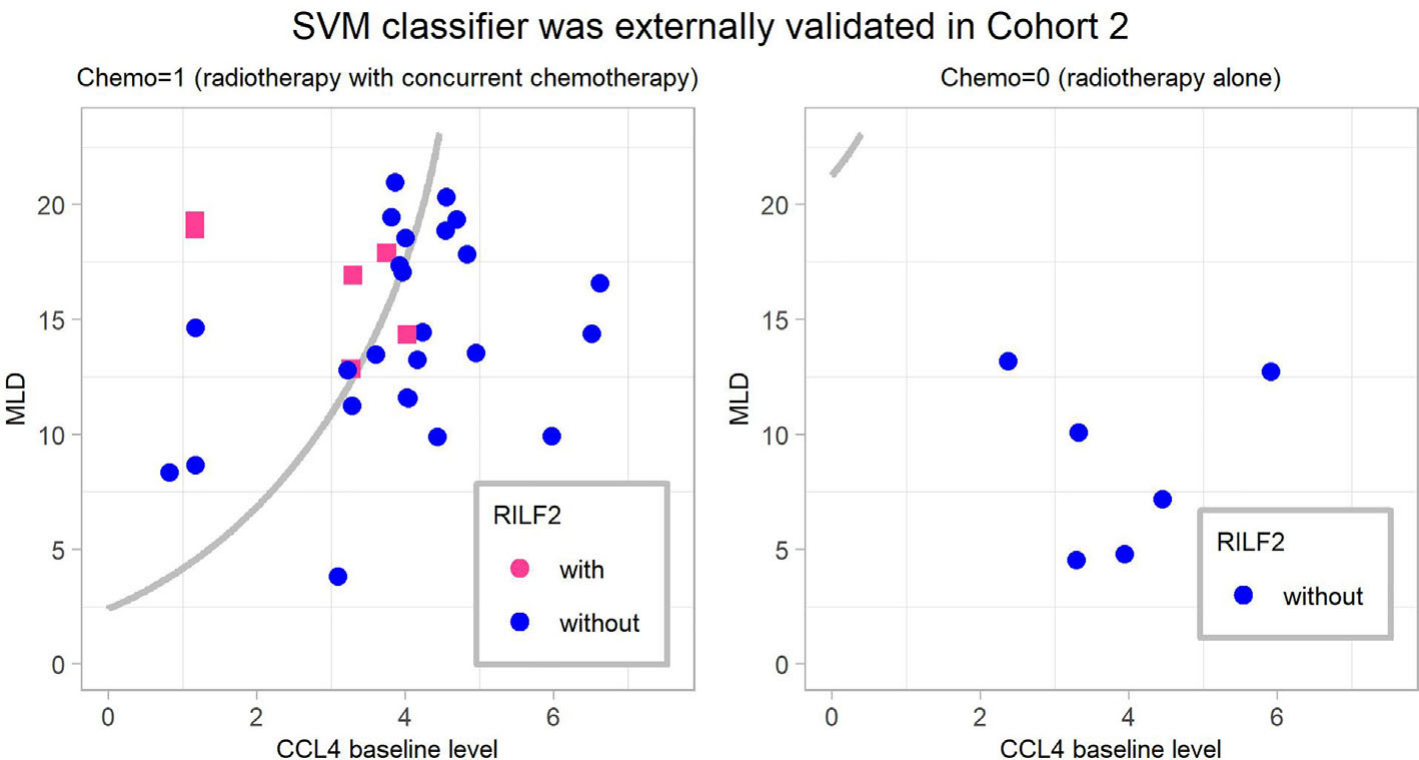
The final weighted-SVM classifier’ external validation in Cohort 2. The cases in the upper-left region of the decision boundary *i.e.* the gray line were predicted occurrence of RILF2 while cases in the bottom-right region were predicted without occurrence of RILF2. The red cases are the real cases with RILF2, and the blue cases are the real cases without RILF2. SVM, Support Vector Machine.

The final SVM model was externally validated in Cohort 2. It predicted RILF2 risk with accuracy = 0.757, sensitivity = 0.833 specificity = 0.742, PPV = 0.385, NPV = 0.958, AUC = 0.855 (95%confidence interval, CI=0.712–0.998, *P* < 0.001), PRAUC = 0.595. The validated results are shown in **Figure 4**; six patients who developed RILF2 are shown as red dots, five patients were predicted to have RILF2 risk (sensitivity = 0.833); while 31 patients without RILF2 are shown as blue dots, 23 patients were predicted to be without RILF2 risk, while eight patients were misjudged (specificity = 0.742). Alternatively, a total of 13 patients in the region to the upper-left boundary were predicted to have RILF2, five patients actually had RILF2 risk (PPV = 0.385), while in the region to the down-right boundary, a total 24 patients were not predicted to have RILF2, 23 patients did not have RILF2 (NPV = 0.958).

**Figure 5:**
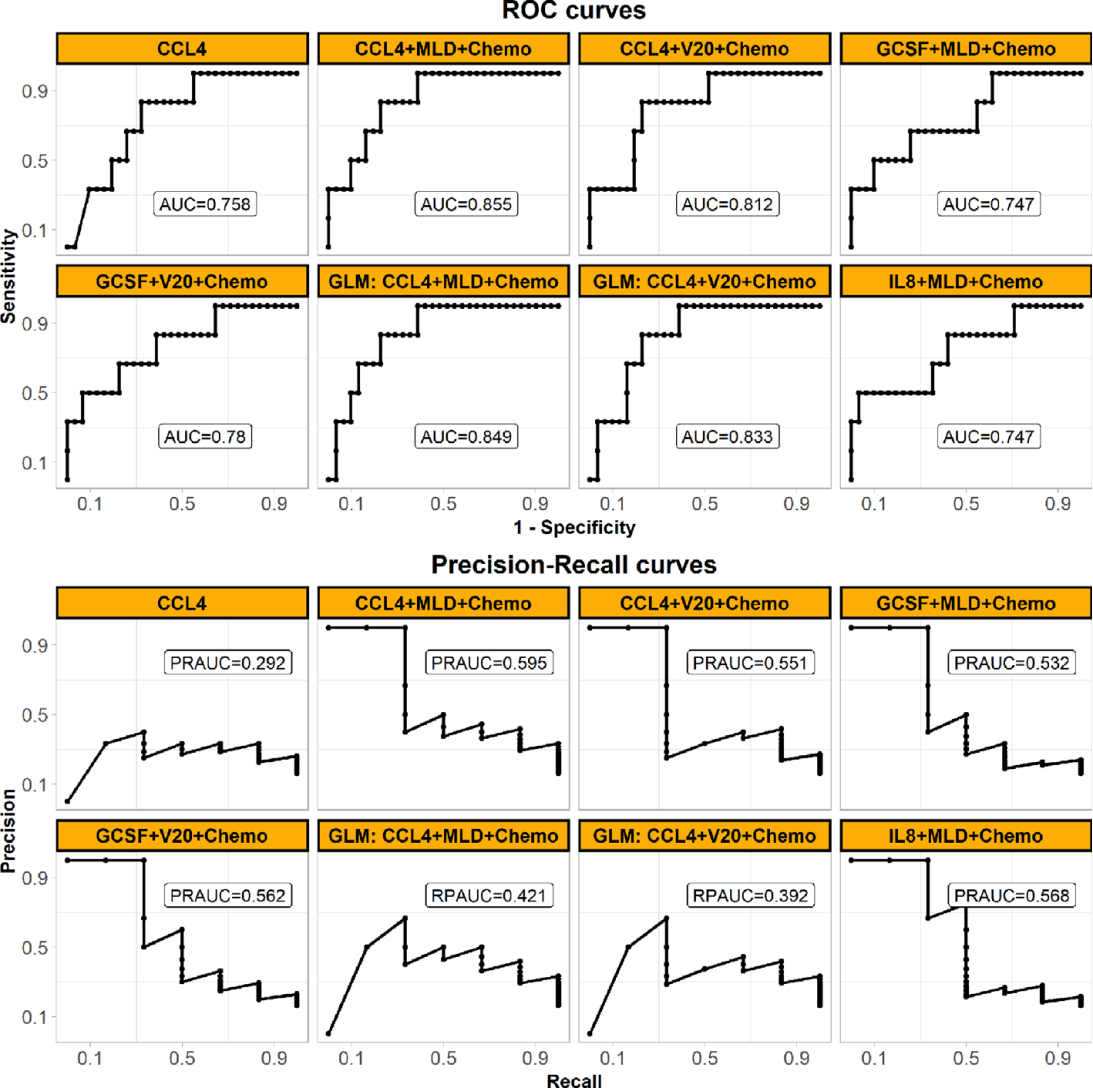
Prediction performances of the final weighted-SVM classifier models externally validated in Cohort 2, comparing with the other models. The panel shows ROC (upper) and precision-recall curves (bottom) performances in the external validation Cohort 2. All models are applied weighted-SVM algorithm except the models applied GLM algorithm (also called logistic regression here) as shown in subplots’ head with “GLM”. The models’ features are also shown in subplots’ head. The panel demonstrates that the final model of using CCL4, MLD, and Chemo with the best generalized performance. (Abbreviations: ROC, receiver operating characteristic; MLD, mean lung dose; Chemo, concurrent chemoradiotherapy; AUC, the area under the ROC curve; PRAUC, the area under the precision-recall curve; GLM, generalized linear model).

The performances of the final SVM classifier were compared with that of conventional GLM models (also built in Cohort 1 and listed in **Supplemental Table 2**) in Cohort 2 as shown in **Figure 5**. The ROC curve (AUC = 0.855) of the final SVM classifier was not only higher than that of the SVM classifiers with other features of our interest as described above Cohort 1, but also higher than the conventional GLM models. Moreover, considering the imbalanced cases, the PR curve (PRAUC = 0.595) of the final SVM classifier wasn’t close to 1, but it was still remarkably higher than the rate of RILF2 risk (0.162) and also the highest in **Figure 5**, especially comparing with GLM models.

## Discussion

In patients with locally advanced NSCLC treated with predominantly conventionally or slightly hypofractionated radiation therapy, this study demonstrated a significant correlation of the pre-treatment cytokine biomarkers CCL4 and G-CSF with RILF2 risk. Machine learning models integrating CCL4 with dosimetric and clinical parameters for RILF2 risk prediction showed reasonable predictive values. Within the limitations of a moderate sized study, the AUC and PRAUC of the final weighted-SVM model showed reasonable performances for predicting RILF2 risk.

Prior reports have highlighted that machine learning approaches can better predict radiation-induced lung disease (17, 26, 27) due to high model accuracy and diminished overfitting (19). SVM is highly resistant to over-fitting because of their mapping into finite dimensional spaces (19). Furthermore, weighted-SVM algorithms (20, 21) are capable of dealing with datasets with imbalanced class frequencies by changing the misclassification penalty per class. Consistent with this, our data (**Figure 5**) shows that weighted SVM generated classifier had better performances than the conventional GLM model. Moreover, as shown in **Figures 3** and **4**, the SVM classifier may be used as an intuitive and convenient tool to help clinical decision making. For example, a clinician could estimate the risk of RILF by evaluating CCL4 baseline level, MLD, and chemotherapy. Should a patient be estimated to have high risk of RILF2, the physician could modify the radiation treatment plan to decrease the risk of RILF2 by decreasing the MLD.

Although a link between CCL4 and radiation-induced toxicity has been previously unappreciated, CCL4 (also known as MIP-1 beta) has been reported to be elevated in the bronchiolar lavage fluid of patients with lung fibrosis as compared to healthy controls (28). It also has been suggested that CCL4 levels are elevated in patients with idiopathic pulmonary fibrosis (29). Ishida Y et al. (30) have found that CCL3, another chemoattractant for CCR5-expressing cells the same as CCL4, was enhanced rapidly and remained at elevated levels after injection bleomycin into wild-type mice until fibrosis developed. But the cytokine milieu which predisposes to radiotherapy-induced fibrosis is not well understood. On the other hand, this study also demonstrated the significance of the pre-treatment levels of other cytokines such as G-CSF with RILF2 in NSCLC patients though they were not into the SVM model achieved by the machine learning algorithm. In mice that received a high-dose G-CSF for 7 consecutive days right after autologous fat grafting, high-dose G-CSF injection was found to have a prolonged macrophage infiltration and elevated levels of inflammation, which could be the direct cause of severe fibrosis (31). Future studies are needed to define the contribution CLL4 and G-CSF to RILF as well external validate our results.

Interestingly, our results suggest that high baseline levels of CCL4 and G-CSF were associated with lower RILF risk, while elevations in these cytokines promoted inflammation and fibrosis in some previous reports in fibrosis with inclusion of animal studies (30). While the reason of this is unclear, such inconsistencies are not uncommon, particularly in comparison between animal and human. For example, cytokine IL-8 induced collagen synthesis and cell proliferation (32) in animal studies though high levels of IL-8 were often found to have an anti-inflammatory effect in human studies (33, 34) and also as shown in our previous work in RP (17). It is also known that increased concentration of cytokines, such as IL-8, CCL4, and G-CSF, might influence macrophages, neutrophils, and lymphocytes chemotaxis and promote pneumonitis and subsequent fibrosis formation. In a study of cutaneous systemic sclerosis patients, CCL4 was augmented along with elevation of myeloid dendritic cells in patients with lung fibrosis (35). One has to note that there was no published study to our knowledge that focused on the role of CCL4 on RILF. It is possible that patients with lower baseline levels of CCL4 might be more sensitive to radiation damage, thus more susceptible to the formation of fibrosis after radiation therapy. On the other hand, high baseline levels of CCL4 may act like against the formation of fibrosis. This matches the results of low PPV value and high NPV value. The exact role of CCL4 needs to be tested in future studies.

It is encouraging to note that SVM classifier which integrated MLD and chemotherapy improved predictive accuracy. This finding was consistent with previous studies (8, 12–14). In our study, the correlation of MLD with RILF2 risk was not significant on univariate analysis as shown in **Table 2**, but it was still an important feature in the final SVM classifier. In **Figure 3** of the SVM classifier, it can be seen that the patients, who with low CCL4 baseline levels were classified to have high risk of RILF2 when MLD was high. MLD is an important radiation dosimetric factor which is normally limited during RT planning. the clinical integration of this SVM classifier may assist in evaluation of radiation plans and enable selection of the optimal plan with the lowest RILF2 risk for each individual patient. Finally, it is interesting that RILF2 was not observed in patients treated with radiotherapy alone in these two cohorts, albeit with limited sample size. The mechanism remains unclear but may be related to the selection criteria and the effects of concurrent chemotherapy with radiation therapy by both increasing tissue injury and altering immune responses.

Of additional note, previous studies have highlighted that the GTV is predictive of lung fibrosis when using SBRT (36). The conventional statistical testing (**Table 2**) also showed GTV with some trend of association with RILF2 (P = 0.07 in Cohort 1), GTV was also considered in the Weighted-SVM classification process as the clinical variable. GTV was not included in the final model in the machine learning framework as receipt of chemotherapy better informed prediction and we restricted our model to one clinical feature to prevent overfitting. GTV may have been less predictive in our dataset because its impact was already being taken into consideration in the lung dose metrics (MLD or V20), and it can’t present the various doses/fractions in this study.

There are some limitations of this study. First, the sample size is small which limits statistical evaluation and constraints machine learning model selection. Second, the three-factor weighted-SVM was constructed to avoid overfitting but is susceptible to type I error and underfitting. This approach does not include other different significant cytokines in models and thus prevented more in-depth pathway analysis. Third, patients in this study were from four prospective studies and some patients received alternative dose/fractionation schemes necessitating 2 Gy equivalent dose calculation for calibration. Fourth, limited by the number of events, the model in this study simplified RILF as a binary outcome, while RILF is a time-dependent five-level graded event which is a topic of our ongoing study. Additionally, the patients were somewhat heterogenous with dose per fraction and biological effective doses were computed with an assumed alpha-beta ratio which could be sources of potential bias. These limitations could be addressed in future validation studies with larger sample sizes.

In summary, using a machine learning framework, a weighted-SVM classifier of RILF2 risk was established which integrated CCL4, MLD, and chemotherapy as representative of cytokines, dosimetric and clinical variables. The weighted-SVM classifier was externally validated and confirmed to have reliable predictive performance. Additionally, our study provides important insights into biomarkers of RILF2 risk and has identified pre-treatment cytokine levels such as CCL4 and G-CSF to be significantly lower in patients who subsequently develop RILF2. Finally, for each individual patient, MLD can be fine-tuned with considering the risk of RILF based on the SVM classifier model. Further study will need to validate this finding and will need to consider the incorporation of other biologic factors such as individual variations of radiation sensitivity to improve positive predictive value for RILF2.

## Reporting Checklist 

The authors have completed the STARD reporting checklist.

## Data Availability Statement

The original contributions presented in the study are included in the article/**Supplementary Material**. Further inquiries can be directed to the corresponding author.

## Ethics Statement

The studies involving human participants were reviewed and approved by Ethics Committee, University of Michigan Health System. The patients/participants provided their written informed consent to participate in this study.

## Author Contributions

(I) Conception and design: HY, CH, MSK. (II) Administrative support: HY, MSK. (III) Provision of study materials or patients: MG, WW, J-yJ, SJ, MSK. (IV) Collection and assembly of data: MG, WW, J-yJ, SJ. (V) Data analysis and interpretation: HY, K-OL, HW, YW. (VI) Manuscript writing: All authors. All authors contributed to the article and approved the submitted version.

## Funding

This project was supported in parts by Shenzhen Fundamental Research Program (No. JCYJ2020109150427184), Shenzhen Science and Technology Program (No.KQTD20180411185028798) and Shenzhen Fundamental Research Program (No.JCYJ20180508153249223). The funders had no role in the initiation or design of the study, collection of samples, analysis, interpretation of data, writing of the paper, or the submission for publication. The study and researchers are independent of the funders.

## Conflict of Interest

The authors declare that the research was conducted in the absence of any commercial or financial relationships that could be construed as a potential conflict of interest.
