# Supplementary material for: Weighted-Support Vector Machine Learning Classifier of Circulating Cytokine Biomarkers to Predict Radiation-Induced Lung Fibrosis in Non-Small-Cell Lung Cancer Patients
Source: Front Oncol. 2021 Feb 1;10:601979. doi: 10.3389/fonc.2020.601979 (PMC7883680; doi:10.3389/fonc.2020.601979)
Supplement: Supplementary file 1 [file Table_1.docx]

| **Table S1. Detail of Four Prospective Clinical Trials.** | | | |
| --- | --- | --- | --- |
| **Clinical Trials** | **Title** | **Radiotherapy** | **Chemotherapy** |
| **2003.073**  **(NCT: NA)** | A Phase I/II Randomized Trial in Radiation Dose Escalation and Timing of Concurrent Chemotherapy for Patients with Stage III Unresectable/ Inoperable Non-Small Cell Lung Cancer | 2 Gy Monday through Wednesday  2.35-3.8 Gy Thursday and Friday for 6 weeks  ≤ 15% NTCP for grade ≥ 3 pneumonitis (EQD2 between 64 and 86 Gy in 30 fractions) | Paclitaxel 45 mg/m^2^  Carboplatin AUC 2 |
| **2003.076**  **(NCT: NA)** | A Pilot Study to Evaluate the Impact of Multiple Functional Images and Molecular Markers on Radiation Treatment Planning and Radiation Outcome and Lung Toxicity Prediction | Determined by radiation oncologist (EQD2 between 64 and 86 Gy in 30 fractions) | Per treating physician |
| **2006.040 (NCT00603057)** | Using Functional Image and Circulating Molecular Markers to Predict Tumor Response and Lung Toxicity in Treatment of Lung Cancer | Determined by radiation oncologist (EQD2 between 60 and 96 Gy in 30 fractions) | Per treating physician |
| **2007.123 (NCT01190527)** | Using FDG-PET Acquired During the Course of Radiation Therapy to Individualize Adaptive Radiation Dose Escalation in Patients with Non-Small Cell Lung Cancer | 2.1-2.85 Gy per day  ≤17.2% NTCP for normal lung (EQD2 between 64 and 92 Gy in 30 fractions) | Carboplatin AUC 2 |
