# Supplementary material for: Weighted-Support Vector Machine Learning Classifier of Circulating Cytokine Biomarkers to Predict Radiation-Induced Lung Fibrosis in Non-Small-Cell Lung Cancer Patients
Source: Front Oncol. 2021 Feb 1;10:601979. doi: 10.3389/fonc.2020.601979 (PMC7883680; doi:10.3389/fonc.2020.601979)
Supplement: Supplementary file 2 [file Table_2.docx]

| Supplemental Table 2. The results of logistic regression models for comparison | | | | | |
| --- | --- | --- | --- | --- | --- |
| variables |  | Estimate coefficient | Standard error | Odds ratio ^a^  (95% confidence interval) | *P* |
| GLM model1 | (Intercept) | -14.921 | 2804.602 | 0 (NA) | 1 |
|  | CCL4 | -0.916 | 0.372 | 0.4 (0.172 - 0.776) | 0.014 * |
|  | MLD | -0.151 | 0.196 | 0.86 (0.57 - 1.263) | 0.441 |
|  | Chemo | 18.837 | 2804.602 | 1.52e8 (0 - Inf) | 1 |
| GLM model2 | (Intercept) | -15.529 | 2678.827 | 0 (NA) | 1 |
|  | CCL4 | -0.991 | 0.372 | 0.371 (0.159 - 0.713) | 0.007 * |
|  | V20 | -0.034 | 0.104 | 0.966 (0.772 - 1.176) | 0.739 |
|  | Chemo | 18.029 | 2678.827 | 6.765e8 (0 - Inf) | 1 |

a.Ratio of levels in patient with RILF2/without RILF2.

* P value < 0.05
